# Establishment of Local Diagnostic Reference Levels for Head CT Imaging in the Madina Region, Saudi Arabia

**DOI:** 10.3390/diagnostics14242882

**Published:** 2024-12-21

**Authors:** Sultan F. Alhujaili, Abdulaziz S. Alshabibi, Feras Alafer, Ziyad Alrowaili, Hasan Salah, Abdelmoneim Sulieman, Mahmoud Subahi, Khaled Al-Raddadi, Mohamed Abuzaid

**Affiliations:** 1 Radiation Sciences and Medical Imaging Department, College of Applied Medical Sciences, Jouf University, Sakaka 72388, Saudi Arabia; sfalhujaili@ju.edu.sa (S.F.A.);; 2 Department of Radiological Sciences, The College of Applied Medical Sciences, King Saud University, Riyadh 12372, Saudi Arabia; 3 Physics Department, College of Science, Jouf University, P.O. Box 2014, Sakaka 72388, Saudi Arabia; 4 Nuclear Medicine Department, Inaya Medical College, Riyadh 13541, Saudi Arabia; 5 Radiology Sciences Department, College of Applied Medical Sciences, King Saud Bin Abdulaziz University Health Sciences, P.O. Box 2477, Al-Ahsa 31982, Saudi Arabia; 6 Department of Radiology, King Salman Medical City, Ministry of Health, Madina 42319, Saudi Arabia; 7 King Fahad Hospital, Ministry of Health, Madina 42351, Saudi Arabia; 8 Medical Diagnostic Imaging Department, College of Health Science, University of Sharjah, Sharjah 27272, United Arab Emirates

**Keywords:** diagnostic reference levels, computed tomography, head CT, radiation exposure, Saudi Arabia

## Abstract

**Background**: Computed Tomography (CT) is crucial in medical diagnosis, particularly for head examinations. Diagnostic Reference Levels (DRLs) are pivotal in balancing diagnostic efficacy with radiation safety. International organizations such as the International Atomic Energy Agency (IAEA) and the International Commission on Radiological Protection (ICRP) provide guidelines for establishing DRLs, emphasizing their importance in optimizing radiation doses. **Aim**: This study aimed to establish and standardize DRLs for head CT imaging in institutional and regional settings, emphasizing the need for tailored reference levels specific to each region’s practices and standards. **Method**: Data collection included patient demographics, imaging parameters, and radiation dose indices, namely volume-weighted CT dose index (CTDIvol) and dose-length product (DLP). Statistical analyses were conducted to determine the median and 3rd quartile values for establishing DRLs. The results were compared with national and international benchmarks to assess variations in radiation doses across regions. **Results**: Demographic profiles detailed gender distributions and ages across hospitals. Imaging parameters exhibited variability in tube voltage, milliampere-seconds (mAs), pitch, scan length, and field of view (FOV). For CTDIvol, the 3rd quartile value was 65.8 mGy (range: 24.8–85.9 mGy), and for DLP, it was 1230.95 mGy·cm (range: 382.3–1189.0 mGy·cm). These values were slightly higher than the national DRLs for Saudi Arabia in 2021 and other international benchmarks, underscoring the need for further optimization and alignment of protocols. **Conclusion**: Optimizing and standardizing DRLs for head CT imaging is crucial for effectively managing radiation doses while ensuring diagnostic accuracy. Comparison with national and international benchmarks highlighted the importance of tailoring reference levels to regional practices and standards, ensuring patient safety without compromising diagnostic efficacy.

## 1. Introduction

Computed tomography (CT) has transformed medical imaging, providing detailed insights, especially for brain conditions. However, the radiation exposure from CT scans poses cancer risks, making the management of this exposure crucial in healthcare [1]. To address radiation dose, Diagnostic Reference Levels (DRLs) are recommended as benchmarks to identify and reduce radiation doses, ensuring patient safety while maintaining diagnostic quality under the ALARA (As Low As Reasonably Achievable) principle.

DRLs play a crucial role in radiation protection by providing a reference point for the radiation doses administered during various imaging procedures. They are typically set at the 75th percentile of the dose distribution observed in a large sample of patients, meaning that 75% of the doses should be below the DRL [1,2,3]. This approach allows for identifying outliers, where doses may be unjustifiably and unnecessarily high. When doses exceed the DRL without any valid reason, it prompts a review of imaging protocols and practices to identify opportunities for dose optimization.

The International Atomic Energy Agency (IAEA) and the International Commission on Radiological Protection (ICRP) have been instrumental in promoting the use of DRLs [4]. These organizations provide guidelines and recommendations to help countries establish and implement DRLs in medical imaging. The ICRP emphasizes the importance of DRLs as part of a broader radiation protection strategy, which includes justification of imaging procedures and optimization of imaging protocols. The ICRP, on the other hand, provides scientific recommendations on radiation protection, including using DRLs as a tool for dose management [1,2,3,4].

While DRLs are widely recognized, their implementation varies across regions due to differences in healthcare practices, equipment, patient demographics, and regulatory frameworks. For example, the DRLs established in one country may not be directly applicable in another due to variations in typical patient populations, imaging equipment, and prevalent medical conditions. Therefore, many countries develop national DRLs based on local data tailored to their specific needs, ensuring that these benchmarks are relevant and effective.

These national DRLs are often compared to international benchmarks to verify that they align with global standards, even though they are customized to the local context. Countries with advanced radiation protection programs, like the United Kingdom and Japan [5], often set DRLs that serve as reference points for others developing their own standards. Given the variability in imaging practices and patient demographics, there is a strong need for localized DRLs that accurately reflect a region or country’s specific conditions and practices.

This is especially important in regions where healthcare practices differ from those in countries where the international DRLs were initially developed. In such cases, localized DRLs ensure that radiation protection measures are relevant and effective locally. Establishing a DRL involves collecting and analyzing radiation dose data from a representative sample of imaging procedures [1,6]. Moreover, establishing DRLs is not a one-time process; it requires regular review and adjustment to account for technological advances, clinical practice changes, and patient demographic shifts. This ongoing process ensures that DRLs remain current and continue to serve their purpose of optimizing radiation safety while maintaining high diagnostic quality. By continually refining these benchmarks, healthcare providers can better manage radiation doses, reducing unnecessary exposure and enhancing patient safety across diverse healthcare settings.

In Saudi Arabia, efforts have been made to establish DRLs for various imaging procedures, including CT scans. However, these DRLs need continuous evaluation and optimization to remain relevant and effective. The Madina region of Saudi Arabia presents a unique case for the establishment of localized DRLs for head CT imaging. The region’s healthcare facilities vary in terms of equipment and imaging protocols, necessitating a tailored approach to radiation protection.

Research on Diagnostic Reference Levels (DRLs) in Saudi Arabia has revealed variations in radiation doses across different regions and hospitals. For instance, studies indicate that DRLs for CT scans in some parts of the country exceed international benchmarks, highlighting the need for dose optimization [7]. CT head examinations are among the most frequently performed CT procedures and involve higher radiation doses than other imaging modalities. These factors make them a priority for establishing DRLs, as optimizing doses for such widely used and high-dose procedures significantly enhances patient safety and supports standardized imaging practices. This study addresses these variations in the Madina region by establishing localized DRLs for head CT imaging tailored to the region’s healthcare practices and patient demographics. By analyzing radiation dose data, the study seeks to create region-specific benchmarks aligning with global standards, promoting standardized practices and ensuring radiation safety and diagnostic accuracy.

## 2. Materials and Methods

### 2.1. Demographics and Facility Selection

This study was conducted across the four hospitals in the Al Madina Region of Saudi Arabia, involving seven distinct CT machines. CT scanners in the study met the inclusion criteria of being registered with the regulatory authority, passing regular quality control (QC) checks and maintenance, and performing more than 100 CT head procedures monthly. Additionally, participating hospitals were required to agree to provide complete and accurate data sets. The study focused exclusively on adult patients (18 years and older) undergoing routine head CT scans, specifically neurocranium/brain scans, for diagnostic purposes. Exclusion criteria included CT scanners undergoing maintenance or those not operational during the data collection period and non-routine head CT scans, such as sinusitis or trauma scans involving the midface, jaws, and upper cervical spine. Pediatric patients (under 18 years old) and cases with incomplete dose or essential parameter data were excluded to ensure consistency and focus on routine procedures. These criteria were designed to maintain the reliability and uniformity of the data collected for establishing Diagnostic Reference Levels (DRLs). The hospitals included in the study were anonymized as Hospital A, Hospital B, Hospital C, and Hospital D. The CT machines at these facilities included models from General Electric, Canon, and Siemens, as detailed in Table 1. The data collection process was conducted retrospectively, focusing exclusively on CT examinations performed during the same period, from May to August 2023. Only CT scanners registered with the regulatory authority and performing more than 100 CT procedures per month were included, focusing specifically on routine head CT scans (neurocranium/brain) with and without contrast. Adult patients (18 years and older) undergoing these routine head CT scans for diagnostic purposes were eligible, and only hospitals in the Madina region that agreed to participate and provide complete data sets were included.

### 2.2. Study Design and Data Collection

This study specifically focuses on neurocranium/brain CT scans (CCT), commonly used in diagnosing conditions related to the brain. Other head CT scans, such as sinusitis scans and head trauma, were excluded due to their different scan ranges.

The study design is based on a previously validated data collection tool [2,3]. The data collected detailed information on CT scanners, routine CT protocols, patient demographics (age, gender), and key dose indices, such as CT dose index volume (CTDIvol) and dose length product (DLP). Data were gathered by selected personnel at each facility, all experienced in CT operations and had undergone a brief training session on the importance of high-quality data collection.

Hospitals were allotted 16 weeks to complete the survey, which collected data from 20 patients per procedure to ensure a reliable representation of typical practices. The results were compiled using standardized MS Excel templates, and all data were carefully checked for errors before analysis.

All scanners underwent calibration and quality control (QC) programs established in the hospitals, as required by local regulations, which include regular traceable calibration of the CT scanners to ensure compliance with dose accuracy standards. To ensure consistency in data collection across multiple hospitals, the study utilized standardized data collection protocols and Microsoft Excel templates, ensuring uniformity in the metrics collected. Personnel at each hospital were trained in proper data collection methods, and error checking involved verifying data completeness, identifying outliers, and cross-referencing entries with hospital records for accuracy. The decision to use the median and 3rd quartile values was based on ICRP 135 guidelines, which recommend the 75th percentile for establishing DRLs to represent typical practices. These measures ensured consistent and accurate data collection across all participating facilities.

### 2.3. Statistical Analysis

The collected data were organized and analyzed using Microsoft Excel and SPSS v. 23.0 (PASW, Chicago, IL, USA). The analysis focused on CTDIvol and DLP. Key statistical parameters were calculated to understand the data distribution, including minimum, maximum, mean, standard deviation, and quartiles (25th, 50th, and 75th percentiles). The median values of CTDIvol and DLP were used to propose achievable doses, while the third quartile values were used to establish Diagnostic Reference Levels (DRLs) [2,3,8]. These DRLs were compared with national and international benchmarks to ensure alignment with established standards. National benchmarks included the Saudi DRLs from 2021, 2020, and 2016. International benchmarks used for comparison included DRLs from Brazil (2023), the UK (2022), Uganda (2022), Italy (2020), Nigeria (2018), Libya (2018), and Japan (2017). 

### 2.4. Ethical Considerations

The study was approved by the institutional review board, the general directorate of health affairs in Madinah, with NCBE-KACST, KSA: (h-03-M-84).

## 3. Results

### 3.1. Demographic Profile of Patients Undergoing CT Scans

Table 2 details gender distribution and average ages of patients across diverse hospitals and CT machine types during CT scans. For instance, (A) (GE) had 22 males (57.9%) and 16 females (42.1%), averaging 39.08 years ± 17.9. (A) (Canon) showed 14 males (36.8%) and 24 females (63.2%), averaging 51.8 years ± 21.7. (A) (GE.BS) had 22 males (57.9%) and 16 females (42.1%), averaging 44.2 years ± 20.8. (B) (S.S40) displayed 15 males (50.0%) and 15 females (50.0%), averaging 48.1 years ± 25.8. (C) (Canon 128) and (C) (Canon 64) depicted an equal split of males and females, averaging 55.9 years ± 20.5 and 50.7 years ± 23.0, respectively. (D) (S. Emotion 6) had 24 males (63.2%) and 14 females (36.8%).

### 3.2. CT Imaging Parameters

Table 3 presents various CT imaging parameters, including tube voltage (KV), mAs (milliampere-seconds), pitch, scan length (in millimeters), and field of view (FOV) for CT machines across different hospitals and models. The values are represented as mean ± standard deviation (SD), indicating the average and the extent of variability within the dataset. The table overviews key CT imaging parameters across various machines and models in multiple hospitals. Tube Voltage (KV) indicates a consistent average of 120 ± 0 KV for most machines, except for (D) (S. Emotion 6) at 126.8 ± 7.3 KV. mAs (Milliampere-Seconds) demonstrates variability, ranging from 196.8 ± 9.2 to 384.8 ± 869.3, reflecting diverse settings impacting radiation exposure and image quality. The pitch values exhibit variation, mostly at 0.5 ± 0 or 0.8 ± 0, with specific instances like 43.9 ± 4.5 for (A) (Canon). Scan length (mm) varies across machines, ranging from 148 ± 9.5 to 190.3 ± 29 mm. Field of view (FOV) varies, with most machines around 223.2 ± 13.9 or 250 ± 0, except for specific models like (A) (Canon) at 219.6 ± 6 and (A) (GE.BS) at 233.4 ± 1.5. These variations underscore the diversity in imaging protocols and practices across the CT machines and models in different hospital settings. A correlation analysis between scan length and DLP revealed a strong positive relationship, with a correlation coefficient of approximately 0.84. This indicates that variations in scan length significantly contribute to the observed variations in DLP. These findings highlight the importance of optimizing scan protocols, particularly by minimizing scan lengths to the necessary anatomical regions to reduce unnecessary radiation exposure while maintaining diagnostic quality.

### 3.3. CTDI Measurements Across CT Machines and Hospitals

Table 4 presents CTDIvol (Computed Tomography Dose Index volume) data from various CT machines across different hospitals, featuring mean values with standard deviation (SD) and quartile representations. The analysis highlights the following distinctive patterns:

- (A) (GE) and (B) (S.S40) exhibit relatively consistent CTDIvol measurements, showing narrower standard deviations and quartile ranges concentrated around their averages. Conversely, (A) (Canon) demonstrates wider variability, with substantial dispersion around the average, reflected in broader quartile ranges.
- Comparing models within the same hospital, such as Canon models at (C) (Canon 128 and 64), reveals moderately consistent CTDIvol values. However, nuanced differences in quartile ranges hint at slight disparities in radiation doses despite similarities in manufacturer models.

The findings reveal a diverse spectrum of CTDIvol measurements across CT machines, emphasizing variations in radiation exposure levels during scans. This underscores the consistency and variability among different machines and hospitals, signifying the complexity of managing radiation doses in CT imaging.

### 3.4. DLP Variability Among Different CT Machines and Hospital Settings

Table 5 presents DLP (Dose-Length Product) measurements from various CT machines in different hospitals, indicating average values as mean ± standard deviation and quartile representations.

The DLP measurements exhibit the following diverse patterns among the machines:

- (A) (GE) shows relatively consistent DLP values, concentrated around the average with a narrow standard deviation and quartile range.
- (A) (Canon) displays substantial variability, resulting in a broader range of measurements.
- Machines like (B) (S.S40) and (D) (S. Emotion 6) exhibit consistent DLP measurements with quartile ranges suggesting a focused spread around the average.
- Canon models at (C) show moderately consistent DLP values but with differences between Canon 128 and 64, highlighting disparities in radiation doses within the same hospital.

These findings reveal considerable variability in DLP measurements across CT machines and hospitals, as demonstrated by the wide range and distribution of values observed in the descriptive analysis, including the 1st, median, and 3rd quartile. This variability highlights the importance of standardizing imaging protocols to enhance patient safety, minimize unnecessary radiation exposure, and optimize imaging parameters for consistent DLP measurements across different machines and institutions. The term “variability” here refers to practical differences rather than statistical, focusing on uniformity in clinical practices.

The analysis revealed a strong positive linear relationship between CTDIvol and DLP, with a Pearson correlation coefficient of 0.9358 (*p* < 0.0001), indicating statistical significance. This result confirms that as CTDIvol increases, DLP also increases significantly, aligning with theoretical expectations, as DLP depends on CTDIvol and scan length. The analysis shows that CTDIvol is a strong predictor of DLP, with a very high correlation between the two variables (R = 0.914) and a substantial portion of the variation in DLP explained by CTDIvol (R^2^ = 0.836). The relationship is statistically significant (*p* < 0.001), where for every unit increase in CTDIvol, DLP increases by approximately 9.33 units. [DLP = 587.794 + (9.329 × CTDIvol)].

### 3.5. Establishment of DRLs: Hospital and Regional Benchmarks

The hospital’s Diagnostic Reference Levels (DRLs) for CTDIvol and DLP are established based on the median values (2nd Quartile) derived from the hospital’s data “Table 4 and Table 5”. Meanwhile, the regional DRLs are set differently; the regional CTDIvol DRL is determined as 51.8, while the DLP regional DRL is set at 838.7. These reference levels serve as benchmarks to guide and regulate radiation doses during CT imaging procedures, ensuring that the doses administered to patients remain within safe and standardized limits within the hospital and the broader regional context.

Table 6 presents the distribution of the Computed Tomography Dose Index volume and Dose-Length Product values across quartiles and their ranges and mean values with standard deviations for head CT scans. The 3rd quartile values for CTDIvol and DLP reflect the upper limit below which 75% of the measured doses fall, suggesting that they could serve as the suggested Diagnostic Reference Levels for the Madina region. For CTDIvol, the 3rd quartile is 65.8, indicating that this value could be set as the DRL to which most head CT scans should conform or strive to be below. Similarly, the 3rd quartile for DLP is 1230.95, which could be established as the DRL for the dose-length product in head CT imaging. Adopting these 3rd quartile figures as the DRLs could help standardize radiation doses across different facilities and reduce patient radiation exposure while maintaining diagnostic efficacy.

Table 7 compares national and international CT Dose Index volume (CTDIvol) and Dose-Length Product (DLP) values for head CT scans. The “Current Study” refers to new data being compared against national standards from previous years in Saudi Arabia and international standards from various countries.

The current study’s value for CTDIvol is 65.8 mGy. This is higher than the National Diagnostic Reference Level (NDRL) for Saudi Arabia in 2021 (45.61 mGy) and the Saudi value in 2020 (55.0 mGy) [9,10,11]. Internationally, this value is higher than the UK’s 2022 standard (47.0 mGy) [12] but lower than Japan’s 2017 standard (85.5 mGy) [5].

Regarding DLP, the current study shows a value of 1231.0 mGy-cm. This is considerably higher than Saudi’s NDRL for 2021 (788 mGy-cm) and other international figures, like the UK’s 2022 value (790.0 mGy-cm) [5,12], but lower than Nigeria’s 2018 value (1999.2 mGy-cm) [13].

**Table 7:** Comparative analysis of national and international CTDIvol and DLP values for head CT scans [2,7,9,11,14,15,16,17,18,19].

	CTDIvol										DLP										
	National				International						National				International						
	Current Study	Saudi 2021	NDRL Saudi 2020	Saudi 2016	Brazil 2023	UK 2022	Uganda 2022	Italy 2020	Nigeria 2018	Japan 2017	Current Study	Saudi 2021	Saudi 2020	Saudi 2016	Brazil 2023	UK 2022	Uganda 2022	Italy 2020	Libya, 2018	Nigeria 2018	Japan 2017
Head	65.8	45.61	55.0	70.8	45.0	47.0	56.0	70.0	61.0	85.5	1231.0	788	1026	1082.9	942.0	790.0	1260.3	1300.0	1999.2	1310.0	1359.8

## 4. Discussion

This study evaluated and aimed to standardize diagnostic reference levels (DRLs) for head CT imaging in Madina, KSA, by analyzing data from multiple CT scanners across various hospitals to establish average radiation doses. The results revealed dose variances among institutions and emphasized the need for dose optimization to enhance patient safety and diagnostic accuracy.

Establishing these DRLs aligns with international norms and promotes systematic dose management and quality control in the clinical setting, ultimately reducing unnecessary radiation exposure and improving radiological protection and patient care in Madina, KSA. No differences in DRL values were observed between CT machines from different manufacturers (GE, Canon, Siemens), as the use of standardized protocols and dose optimization technologies, such as automatic exposure control (AEC), ensured consistency across all machines.

Patient demographic differences, such as age and gender variability, were not expected to impact radiation dose in head CT exams, so no adjustments were made in the analysis. The focus remained on standardizing dose parameters (CTDIvol and DLP) to establish Diagnostic Reference Levels (DRLs) across institutions. Future studies may explore demographic influences in other imaging modalities.

The observed variations in DLP values compared to national and international data may be attributed to differences in scan protocols, equipment calibration, and operator practices. For example, longer scan lengths or the use of additional imaging phases in some institutions could contribute to higher DLP values. Additionally, variations in equipment settings, such as tube current (mAs) and voltage (kVp), as well as the adoption of dose optimization technologies, may differ between institutions and regions. Disparities in adherence to standardized protocols and quality control programs could also play a role. Addressing these factors through enhanced training, equipment upgrades, and protocol standardization is essential to achieving consistency and alignment with national and international benchmarks.

To reduce dose values, it is essential to standardize imaging protocols across all institutions. This includes optimizing scan parameters, such as lowering the tube current (mAs) and adjusting the tube voltage (kVp) based on patient size, while ensuring diagnostic image quality. The use of automatic exposure control (AEC) can further tailor radiation doses to individual patient anatomy, avoiding excessive exposure. Additionally, minimizing scan lengths to cover only the necessary anatomical regions and avoiding multiphase scans unless clinically justified are key strategies for dose reduction.

Adopting advanced imaging technologies, such as iterative reconstruction techniques, can significantly lower radiation doses while maintaining image quality. Regular training for radiologists and technologists on dose optimization principles and adhering to DRLs is equally critical. Implementing robust quality control programs, including regular calibration of CT scanners and monitoring of dose metrics, will ensure consistency and help identify opportunities for further optimization. These strategies collectively contribute to reducing unnecessary radiation exposure and enhancing patient safety.

## 5. Limitation

Our study’s limitations include its focus on a specific geographic region, which may limit the generalizability of the findings to other areas that may use different imaging protocols. Additionally, the sample size could have been expanded to include a wider range of institutions for more comprehensive results.

## 6. Conclusions and Recommendations

This study revealed variability in radiation doses (CTDIvol and DLP) during head CT scans across hospitals in Madina, Saudi Arabia, highlighting the need for standardized imaging protocols to minimize dose variations while maintaining diagnostic efficacy. Key recommendations include adopting dose-reduction strategies such as adjusting tube current (mAs) and voltage (kVp), optimizing scan length, and employing techniques like automatic exposure control (AEC) and iterative reconstruction to reduce radiation exposure.

Establishing DRLs based on 3rd-quartile values provides a starting point for standardizing dose practices. Further refinement using image quality-based criteria or organ-specific dose constraints is necessary. Regular education and training for radiologists and technologists, alongside robust quality control programs with audits and feedback, are essential for consistent protocol adherence and continuous improvement.

It is recommended that DRLs be revised every 3 to 5 years to align with technological advancements and clinical practice changes, ensuring enhanced radiation safety and patient care.

## Figures and Tables

**Table 1:** Specifications of CT machines across four hospitals.

Hospital	CT Manufacture	CT Model	Installation Year
(A)	GE	Discovery CT 750 HD	2013
(A)	Canon	Aspire Aquilion	2020
(A)	GE	Bright Speed (BS)	2012
(B)	SIEMENS	Sensation 40	2010
(C)	CANON	Aquilion prim 128	2019
(C)	CANON	Aquilion prim 64	2019
(D)	SIEMENS	Emotion 6	2014

**Table 2:** Demographic distribution of patients undergoing CT Scans in different hospitals and with different CT machine types.

	Gender (N/%)		Total Number of Patients	Age
	Male	Female		Mean ± SD
(A) (GE)	22 (57.9)	16 (42.1)	38	39.08 ± 17.9
(A) (Canon)	14 (36.8%)	24 (63.2%)	38	51.8 ± 21.7
(A) (GE.BS)	22 (57.9%)	16 (42.1%)	38	44.2 ± 20.8
(B) (S.S40)	15 (50.0%)	15 (50%)	30	48.1 ± 25.8
(C) (Canon 128)	50 (50%)	50 (50%)	100	55.9 ± 20.5
(C) (Canon 64)	53 (53%)	47 (47%)	100	50.7 ± 23.0
(D) (S. Emotion 6)	24 (63.2%)	14 (36.8%)	38	34.7 ± 25.2

**Table 3:** Comparison of CT imaging parameters across multiple hospitals and machine models.

Mean ± SD	(A) (GE)	(A) (Canon)	(A) (GE.BS)	(B) (S.S40)	(C) (Canon 128)	(C) (Canon 64)	(D) (S. Emotion 6)
(KV)	120 ± 0	120 ± 0	120 ± 0	120 ± 0	120 ± 0	120 ± 0	126.8 ± 7.3
mAs	248.8 ± 57.7	253.4 ± 67	211.6 ± 89.8	279.4 ± 50.4	384.8 ± 869.3	373.5 ± 588.8	196.8 ± 9.2
Pitch	0.5 ± 0	43.9 ± 4.5	0.5 ± 0.1	0.4 ± 0.3	0.8 ± 0	0.8 ± 0	1.5 ± 0
Scan length (mm)	168.1 ± 10.7	163.0 ± 10.2	148 ± 9.5	190.3 ± 29	158.1 ± 10.7	158.1 ± 10.7	159.4 ± 28.3
(FOV)	224.8 ± 7	219.6 ± 6	233.4 ± 1.5	250 ± 0	223.2 ± 13.9	223.2 ± 13.9	250 ± 0

**Table 4:** CTDIvol measurements across diverse CT machines in different hospitals: mean, variability, and quartile distribution.

	Hospital	Mean ± SD	1st Quartile	2nd Quartile	3rd Quartile
CDTI vol (mGy)	(A) (GE)	55.8 ± 6.7	49.4	56.7	62.5
	(A) (Canon)	7.5 ± 45	36.1	40.7	46.8
	(A) (GE.BS)	43.8 ± 14.5	22.9	51.3	56.1
	(B) (S.S40)	41.8 ± 2.5	40.8	41.8	43.7
	(C) (Canon 128)	54.3 ± 6	49.0	53.0	58.9
	(C) (Canon 64)	60 ± 6.7	55.3	59.6	66.0
	(D) (S. Emotion 6)	26 ± 4	22.9	22.9	27.9

**Table 5:** DLP measurements across diverse CT machines in different hospitals: mean, variability, and quartile distribution.

	Hospital	Mean ± SD	1st Quartile	2nd Quartile	3rd Quartile
DLP (mG.cm)	(A) (GE)	1038.2 ± 84	997.5	997.5	1075.4
	(A) (Canon)	539.9 ± 525.7	532.6	540.0	544.3
	(A) (GE.BS)	594.7 ± 244.9	356.4	491.9	836.4
	(B) (S.S40)	595.8 ± 71.3	541.9	596.3	651.6
	(C) (Canon 128)	303.1 ± 111.6	196.4	316.4	382.3
	(C) (Canon 64)	1084.7 ± 143.9	1003.4	1100.6	1192.2
	(D) (S. Emotion 6)	1206.9 ± 131	1146.5	1189.0	1189.0

**Table 6:** Diagnostic reference levels based on 3rd quartile values for head CT imaging.

	CTDIvol (mGy)						DLP (mG.cm)				
	Range	1st Quartile	2nd Quartile	3rd Quartile	Mean (SD)		Range	1st Quartile	2nd Quartile	3rd Quartile	Mean (SD)
Head	24.8–85.9	52.025	58	65.8	57.7 ± 0.71		61.4–11,159.6	924.455	1090.3	1230.95	1105.3 ± 767.5

## Data Availability

The data presented in this study are available upon request from the corresponding author due to restrictions outlined in the ethical approval that aim to protect participant confidentiality and adhere to consent agreements of public data sharing.

## Abbreviations

AEC	Automatic Exposure Control
AIDR	Adaptive Iterative Dose Reduction
ALARA	As Low As Reasonably Achievable
ASiR	Adaptive Statistical Iterative Reconstruction
CT	Computed Tomography
CTDIvol	Computed Tomography Dose Index Volume
DLP	Dose Length Product
DRL	Diagnostic Reference Level
IAEA	International Atomic Energy Agency
ICRP	International Commission on Radiological Protection
kVp	Kilovolt Peak
mAs	Milliampere seconds
NDRL	National Diagnostic Reference Level
QC	Quality Control
